# Divergent effects of acute and repeated quetiapine treatment on dopamine neuron activity in normal vs. chronic mild stress induced hypodopaminergic states

**DOI:** 10.1038/s41398-017-0039-9

**Published:** 2017-12-11

**Authors:** Jared L. Moreines, Zoe L. Owrutsky, Kimberly G. Gagnon, Anthony A. Grace

**Affiliations:** 1 Departments of Neuroscience, Psychiatry, and Psychology, Center for Neuroscience, University of Pittsburgh, Pittsburgh, PA 15260 USA; 2 Medical Scientist Training Program, University of Pittsburgh School of Medicine, Pittsburgh, PA 15261 USA; 3 Center for the Neural Basis of Cognition, Carnegie Mellon University, Pittsburgh, PA 15213 USA

## Abstract

Clinical evidence supports the use of second-generation dopamine D2 receptor antagonists (D2RAs) as adjunctive therapy or in some cases monotherapy in patients with depression. However, the mechanism for the clinical antidepressant effect of D2RAs remains unclear. Specifically, given accumulating evidence for decreased ventral tegmental area (VTA) dopamine system function in depression, an antidepressant effect of a medication that is expected to further reduce dopamine system activity seems paradoxical. In the present paper we used electrophysiological single unit recordings of identified VTA dopamine neurons to characterize the impact of acute and repeated administration of the D2RA quetiapine at antidepressant doses in non-stressed rats and those exposed to the chronic mild stress (CMS) rodent depression model, the latter modeling the hypodopaminergic state observed in patients with depression. We found that acute quetiapine increased dopamine neuron population activity in non-stressed rats, but not in CMS-exposed rats. Conversely, repeated quetiapine increased VTA dopamine neuron population activity to normal levels in CMS-exposed rats, but had no persisting effects in non-stressed rats. These data suggest that D2RAs may exert their antidepressant actions via differential effects on the dopamine system in a normal vs. hypoactive state. This explanation is supported by prior studies showing that D2RAs differentially impact the dopamine system in animal models of schizophrenia and normal rats; the present results extend this phenomenon to an animal model of depression. These data highlight the importance of studying medications in the context of animal models of psychiatric disorders as well as normal conditions.

## Introduction

Quetiapine and other second-generation dopamine D2 receptor antagonists (D2RA), e.g., lurasidone, are increasingly popular monotherapy and as an adjunct to other antidepressant drugs in patients with inadequate responses to traditional antidepressants alone^1^. However, the therapeutic mechanism of these agents in depression is largely unknown. For quetiapine specifically, existing theories highlight the role of a drug metabolite *N*-desalkyl quetiapine, which functions as a potent norepinephrine reuptake inhibitor^2^. However, this model does not incorporate the D2RA properties of quetiapine.

Mounting evidence suggests that in depression the dopamine system is underactive^3–6^. Thus, it is important to reconcile how a compound that is thought to block dopamine transmission may actually help a condition that results from already reduced dopamine system function^7^. Importantly, existing theories are based on data from administering quetiapine to normal rats, whereas the effect of quetiapine on the dopamine system in rodent depression models has not been examined. However, as has become clear in other settings such as animal models of schizophrenia, the effects of D2RAs on a normally functioning dopamine system often differ substantially from their effects on a dopamine system functioning in a pathological state^8^. It is therefore critical to examine the impact of quetiapine on animal models of depression that exhibit clinically relevant pathological functioning of the dopamine system.

Dopamine neurons fire in two patterns: a slow, irregular discharge pattern of tonic activity, and a rapid sequential discharge of burst firing^9^. Most dopamine neurons alternate between these two firing patterns, however in order to burst fire a neuron must already be in a spontaneously active state. Using single unit electrophysiological recordings of identified dopamine neurons, one can quantify the relative number of spontaneously active dopamine neurons, as well as the proportion of their firing that occurs during a burst. We have recently reported that across multiple animal models of depression, including chronic mild stress (CMS)^10^ and learned helplessness^11^, the number of spontaneously active dopamine neurons is markedly reduced without changes to their firing rate or amount of burst firing. In the CMS model specifically, we have further elucidated that this decrease in dopamine population activity following exposure to chronic stress is due to enhanced afferent GABA-ergic input from the ventral pallidum driving dopamine neurons into a silent state, as inactivation of this region restores dopamine population activity to normal levels^12^.

Multiple studies across animal models^13^ and human patients with depression^14^ suggest that restoring normal dopamine system function is a valuable approach to treating depression, particularly in cases with prominent dopamine related symptoms such as anhedonia. Thus, if quetiapine were to increase the population activity of dopamine neurons, this would be expected to contribute to its antidepressant effect. While the effects of quetiapine on the dopamine system have been extensively studied in normal rodents, the effects of quetiapine on the dopamine system in animal models of depression have not been examined. Specifically, acute administration of quetiapine at antidepressant doses has been shown to increase dopamine population activity^15^, whereas after repeated administration dopamine neuron population activity is at baseline or lower levels^16^. However, behavioral studies using the CMS model suggest that quetiapine may have the ability to reverse deficits in behavioral assays relevant to depression, specifically when administered at doses of 10 mg/kg for long durations^17^. This dose is comparable to 150–300 mg daily in humans, which has been supported to have antidepressant effects and is substantially lower than the doses used for schizophrenia^18^. This suggests that the effects of repeated quetiapine may differ in the presence of factors driving a hypodopaminergic state, e.g., chronic stress.

In the present study, we evaluated the acute and repeated treatment effects of antidepressant dosage of quetiapine in both non-stressed and CMS-exposed rats. We find that acute and repeated quetiapine administration produces strikingly different effects in non-stressed vs. stressed rodents.

## Materials and methods

### Subjects

All procedures were performed in accordance with the National Institutes of Health Guide for the Care and Use of Laboratory Animals and were approved by the Institutional Animal Care and Use Committee of the University of Pittsburgh.

Adult male Sprague-Dawley rats (Envigo, Indianapolis, Indiana) weighing 300–340 g at baseline were used for all experiments. They were acclimated to the facilities for >7 days, housed in pairs in a temperature (22 °C) and humidity (47%) controlled colony room (lights ON 7:00 a.m.–7:00 p.m.). Food and water were available ad libitum. Sample size was determined by calculating the group size needed to achieve power ≥ 80%. Rats were randomly assigned to CMS or control and drug or vehicle groups.

### CMS procedure

CMS was performed as reported previously^10,12^. CMS exposed rats were single-housed in individual cages within an isolated colony room. Stressors included periodic restricted access to food and water, cage tilt, damp bedding, continuous overnight illumination, intermittent paired housing with an unfamiliar cage-mate, white noise (80–90 dB), stroboscopic lighting, and predator odor, all delivered in the home cage. Each week rats were exposed to 3–4 stressors in a pre-determined order consistent with our prior published studies^10,12,19^.

### Drug preparation

Quetiapine 10 mg/mL was dissolved in 0.3% tartaric acid in physiological saline and neutralized with sodium hydroxide to a final pH of 5-6. This dose (10 mg/kg) was selected based on prior studies showing electrophysiological^15^ and behavioral^17^ effects at this dose. Vehicle treated rats received 1 ml/kg injections of the same solution without the addition of quetiapine; the two solutions were equivalent in pH and temperature. Compared to humans, rats produce markedly lower levels of the norquetiapine metabolite from quetiapine, allowing us to partially dissociate the D2RA effects of quetiapine from the norepinephrine effects of the norquetiapine metabolite^20^.

### Electrophysiological recordings

#### Surgery

Electrophysiological recordings were performed counterbalanced for light phase and experimenter across groups. Rats anesthetized with chloral hydrate (400 mg/kg; i.p.) were placed in a stereotaxic frame (David Kopf Instruments, Tujunga, CA) and maintained at 37 °C using a thermocouple-controlled heating pad (Fine Science Tools, Foster City, CA). To access the VTA, the skull was cleared of skin and fascia, and a partial craniectomy of the skull was performed 5.3–5.7 mm posterior and + 0.6–1.0 mm lateral to bregma.

#### Signal acquisition

Single barrel electrodes were constructed with a vertical electrode puller (Narishige, Tokyo, Japan) using 2 mm diameter borosilicate capillary tubes (World Precision Instruments, Sarasota, FL), and then broken to a target of 6–10 MΩ under microscopic control and filled with a 2 M saline solution with 2% Chicago Sky Blue (Sigma Aldrich). Signals were fed through silver wire to an amplifier (Fintronics, Orange, CT) operated with open filter settings (10 Hz low cutoff, 16 kHz high cutoff, 1000x gain), displayed on an oscilloscope (B&K Precision, Yorba Linda, CA), and stored on a computer running Lab Chart 7 (AD Instruments, Sidney, Australia). Units were recorded when signal-to-noise ratio exceeded 3:1. Electrode integrity was ensured by replacing electrodes and repeating tracks when either (1) no cells with biphasic waveform (either dopamine or non-dopamine) were identified on two consecutive tracks, or (2) an acute obvious change in recorded neuron shape or background noise level suggested damage to the electrode.

#### VTA sampling and dopamine neuron identification

The VTA was sampled over a grid of nine sequential electrode tracks separated by 0.2 mm and arranged in a predetermined pattern to reliably sample across the medial-lateral and anterior-posterior extent of the A10 region^10^. Electrodes were lowered into the VTA using a manual hydraulic microdrive (Kopf Instruments) and dopamine neurons were identified using well-established criteria including location, slow (<12 Hz) and irregular firing pattern, and long duration action potential (>2.2 msec, with onset to bottom of trough > 1.1 msec) with variable shape and biphasic waveform^21,22^. Identified dopamine neurons were recorded for 3 min (1 min minimum), and three parameters of dopamine neuron firing were calculated: (1) the number of spontaneously firing dopamine neurons identified in each track (i.e., population activity averaged over nine tracks), (2) firing rate, and (3) proportion of spikes occurring as burst firing (%SIB) in which the burst onset was defined as two spikes with ≤80 msec interspike interval and termination by >160 msec interspike interval^23^. Additional bursting characteristics historically examined in studies of dopamine neurons were also calculated including burst duration (sec), bursting rate (Hz), number of spikes per burst, intra-burst inter-spike interval, and coefficient of variation. No significant group differences in these parameters were found (see supplemental information Fig. S1, Tables S1–S2).

### Experimental outline

**Fig. 1:**
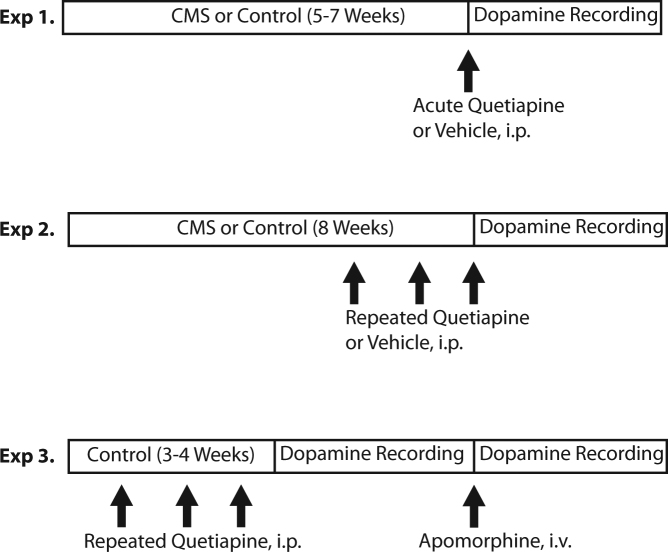
Experimental Timeline. In Experiments 1 and 2, rats experienced 5–7 weeks of CMS or control conditions. This was followed by acute administration of quetiapine on the day of dopamine neuron recording (Experiment 1) or 21+ days of quetiapine with continued CMS exposure up to the day of dopamine neuron recording (Experiment 2). In Experiment 3, rats living in normal housing conditions received 21+ days of quetiapine up to the day of dopamine neuron recording, which included measurements of dopamine neuron population activity before and immediately following a pre-synaptic dose of apomorphine

The experiments performed are outlined in Fig. 1.

*Experiment 1: Acute quetiapine in non-stressed and CMS-exposed rats*. Rats were exposed to 5–7 weeks of CMS or control conditions. On the day of dopamine neuron recording, quetiapine was administered 2 h prior to beginning the recording.

*Experiment 2: Repeated quetiapine in non-stressed and CMS-exposed rats*. Rats were exposed to 5 weeks of standard CMS or control conditions, followed by an additional 3+ weeks during which they received daily injections of quetiapine 10 mg/kg or vehicle in parallel with the CMS protocol. Electrophysiological recordings began 2 h following the final dose.

*Experiment 3: Repeated quetiapine plus apomorphine in non-stressed rats*. Rats received daily injections of quetiapine 10 mg/kg for >21 days, which is a standard minimum duration for the induction of depolarization block^24^. Electrophysiological recordings were performed beginning 2 h after the final dose. A recording of VTA population activity was performed. Apomorphine dissolved in normal saline (50 mcg/mL) was then delivered intravenously at autoreceptor-selective dosages via the lateral tail vein until a decrease in firing rate of >20% was observed, with most rats showing response with 40–80 µg/kg, consistent with prior studies^8^. The VTA was then resampled for population activity. These recordings were counterbalanced across animals for anteroposterior starting location within the VTA.

### Histology

Final electrode placement at the conclusion of recording was marked by electrophoretic ejection of Chicago Sky Blue. Rats were overdosed with additional chloral hydrate and decapitated. Brains were removed and fixed in 8% paraformaldehyde followed by 25% sucrose for cryoprotection. Sixty micrometer coronal sections were prepared using a cryostat (Leica, Buffalo Grove, IL), mounted onto gelatin-chromalum coated glass slides, and stained with cresyl violet and neutral red for microscopic confirmation of recording electrode location. Rats were excluded from the study if histological review showed recording electrode location outside of the VTA. Histological review was conducted with the investigator blinded to an animal’s experimental group.

### Data analysis

Spike time courses for individual neurons were exported from Lab Chart into Neuroexpolorer (Nex Technologies, Madison, AL) for calculation of firing rate and burst firing. Within each rat, population activity was calculated by dividing the total number of dopamine neurons identified by the number of valid tracks performed, yielding the metric of Cells Per Track (CPT). The average firing rate and amount of burst activity for dopamine neurons recorded were averaged across rats in a group, considering each cell as an independent replicate. Data were analyzed in SPSS 23 (IBM, Armonk, NY) and Prism 7 (GraphPad, La Jolla, CA). For experiments 1 and 2, 2-way ANOVAs with post-hoc tests were performed to compare treatment groups. For experiment 3, paired t-tests were performed to compare data collected before and after apomorphine administration.

## Results

### Experiment 1: Acute quetiapine increased dopamine neuron population activity in non-stressed rats but not chronically stressed rats

**Fig. 2:**
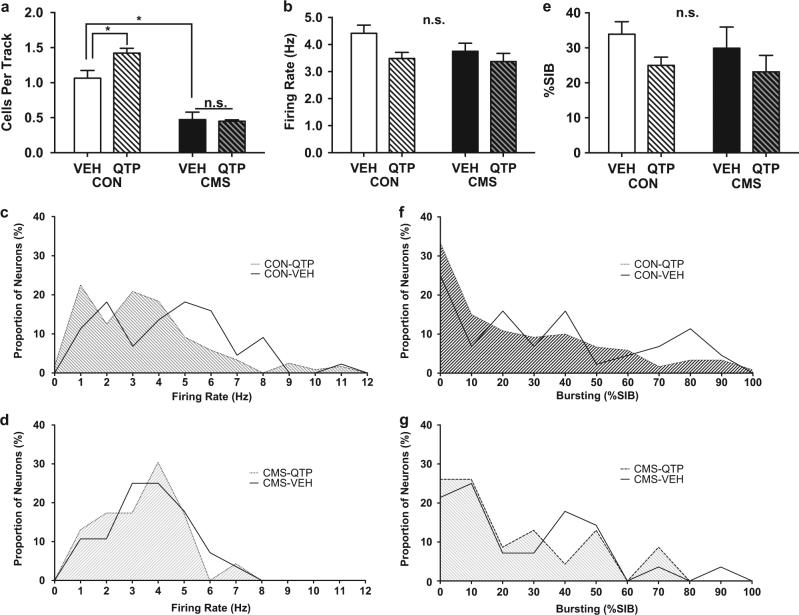
Impact of Acute Quetiapine Treatment in Non-stressed and Chronically Stressed Rats. Acute administration of quetiapine increased dopamine population activity in non-stressed rats, but had no effects on CMS-exposed rats. **a** Dopamine population activity was increased following acute administration of quetiapine to non-stressed rats, but the reduction observed in stressed rats was not ameliorated by acute administration. **b-g** Average dopamine neuron firing rate **b-d** and proportion of burst firing **e-g** were unchanged by acute administration of quetiapine to non-stressed or CMS-exposed rats. The parallel increase in slow firing **c**, nonbursting **f** neurons in non-stressed rats likely reflects activation of previously silent neurons, that when combined with a rightward shift in these parameters in previously firing neurons results in no net change in firing rate or pattern

The differential effect of acute quetiapine in non-stressed and stress-exposed rats was evaluated by administering an acute dose of quetiapine (10 mg/kg) 2 h prior to assessing VTA dopamine neuron firing properties. This resulted in a significant interaction effect between treatment with acute quetiapine and exposure to CMS for dopamine population activity (2-way ANOVA interaction *F*_(1,25)_ = 4.3, *p* = 0.049, Fig. 2, Table 1). Acute administration of quetiapine to non-stressed rats increased dopamine population activity (CON-VEH-Acute 1.1 ± 0.11 CPT, *n* = 8; CON-QTP-Acute 1.4 ± 0.068 CPT, *n* = 11; 2-way ANOVA interaction F_(1,25)_ = 4.3, *p* = 0.049; post-hoc *t*_25_ = 3.3, *p* = 0.0054; Fig. 2a, Table 1), in line with prior reported results^15^. There was no change in the average firing rate of recorded dopamine neurons (CON-VEH-Acute 4.4 ± 0.30 Hz, *n* = 69; CON-QTP-Acute 3.5 ± 0.23 Hz, *n* = 120; 2-way ANOVA *p* > 0.05 for main effects of drug or interaction; Fig. 2b–d, Table 1) or proportion of burst activity (CON-VEH-Acute 33.9 ± 3.6%SIB, *n* = 69; CON-QTP-Acute 25.0 ± 2.4%SIB, *n* = 120; 2-way ANOVA *p* > 0.05 for main effects of drug or interaction; Fig. 2e–g, Table 1). However, histogram examination suggests the increased population activity may involve the emergence of slower firing and less bursting cells, offset by increases in these measures in already active neurons (Fig. 2c, f), resulting in no net change across the population.

**Table 1:** Summary of dopamine neuron data for acute quetiapine treatment in Experiment 1

	CON-VEH-Acute	CON-QTP-Acute	CMS-VEH-Acute	CMS-QTP-Acute
Cells Per Track	**1.1** ± **0.11**	**1.4** ± **0.068**	**0.47** ± **0.11**	**0.45** ± **0.019**
2-Way interaction	**F**_**(1,25)**_ = **4.3;** ***p*** = **0.049** ^**a**^	−	−	−
Drug effect	F_(1,25)_ = 3.3; ***p*** = 0.081^a^	**t**_**(25)**_ = **3.3;** ***p*** = **0.0054** ^**b**^	−	t_(25)_ = 0.15; ***p*** = 0.99^b^
Stress effect	**F**_**(1,25)**_ = **72.6;** ***p*** ** < 0.0001** ^**a**^	−	**t**_**(25)**_ = **4.2;** ***p*** = **0.0006** ^**c**^	**t**_**(25)**_ = **8.3; p < 0.0001** ^**c**^
Firing Rate (Hz)	**4.4** ± **0.30**	**3.5** ± **0.23**	**3.7** ± **0.30**	**3.4** ± **0.31**
2-Way interaction	F_(1,227)_ = 0.46; *p* = 0.50^a^	−^d^	−^d^	−^d^
Drug effect	F_(1,227)_ = 2.6; *p* = 0.11^a^	−^d^	−	−^d^
Stress effect	F_(1,227)_ = 0.94; *p* = 0.33^a^	−	−^d^	−^d^
Bursting (%SIB)	**33.9** ± **3.6**	**25.0** ± **2.4**	**29.9** ± **6.1**	**23.2** ± **4.7**
2-Way interaction	F_(1,227)_ = 0.056; *p* = 0.81^a^	−^d^	−^d^	−^d^
Drug effect	F_(1,227)_ = 2.8; *p* = 0.094^a^	−^d^	−	−^d^
Stress effect	F_(1,277)_ = 0.39; *p* = 0.53^a^	−	−^d^	−^d^
Group N (Rats)	***N*** = **8 rats, 69 cells**	***N*** = **11 rats, 120 cells**	***N*** = **4 rats, 19 cells**	***N*** = **6 rats, 23 cells**

*%SIB*, Percent of Spikes in Burst; *CMS* chronic mild stress, *CON* control, *QTP* quetiapine, *VEH* vehicle;

^a^Main effects among all groups

^b^Sidak’s post-hoc test for drug effect (within same stress category)

^c^Sidak’s post-hoc test for stress effect (within same drug group)

^d^Main effect not significant, post-hoc test not performed

Bold values signify summaries of primary data or a statistically significant analysis of data

In line with prior results, CMS-exposed rats treated acutely with vehicle had fewer spontaneously active dopamine neurons as compared to non-stressed rats treated acutely with vehicle (CON-VEH-Acute 1.1 ± 0.11 CPT, *n* = 8; CMS-VEH-Acute 0.47 ± 0.11 CPT, *n* = 4; 2-way ANOVA main effect of stress *F*_(1,25)_ = 72.6; *p* < 0.0001; post-hoc t_25_ = 4.2, *p* = 0.0006; Fig. 2a, Table 1), without a change in firing rate (CON-VEH-Acute 4.4 ± 0.30 Hz, *n* = 69; CMS-VEH-Acute 3.7 ± 0.30 Hz, *n* = 19; 2-way ANOVA *p* > 0.05 for main effects of stress or interaction; Fig. 2b–d, Table 1) or proportion of burst activity (CON-VEH-Acute 33.9 ± 3.6 %SIB, *n* = 69; CMS-VEH-Acute 29.9 ± 6.1%SIB, *n* = 19; 2-way ANOVA *p* > 0.05 for main effects of stress or interaction; Fig. 2e–g, Table 1).

In contrast to the effects observed in non-stressed rats, quetiapine had no effect on dopamine neuron population activity in CMS-exposed rats (CMS-VEH-Acute 0.47 ± 0.11 CPT, *n* = 4; CMS-QTP-Acute 0.45 ± 0.019 CPT, *n* = 6; post-hoc *t*_25_ = 0.15, *p* = 0.99; Fig. 2a, Table 1). Similarly, there was no effect on average dopamine neuron firing rate (CMS-VEH-Acute 3.7 ± 0.30 Hz, *n* = 19; CMS-QTP-Acute 3.4 ± 0.31 Hz, *n* = 23; 2-way ANOVA *p* > 0.05; Fig. 2b–d, Table 1), or proportion of burst activity (CMS-VEH-Acute 29.9 ± 6.1%SIB, *n* = 19; CMS-QTP-Acute 23.2 ± 4.7%SIB, *n* = 23; 2-way ANOVA *p* > 0.05; Fig. 2e–g, Table 1).

### Experiment 2: Repeated quetiapine normalized decreased dopamine neuron population activity in chronically stressed rats but had no effect in non-stressed rats

**Fig. 3:**
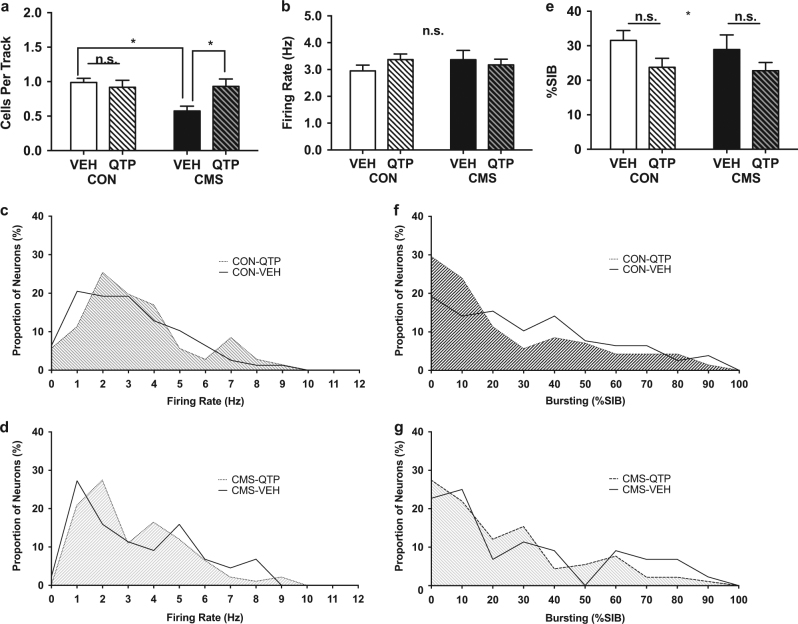
Impact of repeated quetiapine administration in non-stressed and chronically stressed rats. Repeated administration of quetiapine for 21+ days reversed the CMS-induced decrease in dopamine population activity without affecting this measure in non-stressed rats. **a** Dopamine population activity was significantly increased in CMS-exposed rats following repeated quetiapine treatment, but was unchanged in non-stressed rats treated repeatedly with vehicle. **b-d** Average dopamine neuron firing rate was unchanged by chronic stress or quetiapine **b**, nor was the distribution of firing rates **c-d**. **e-g** Despite a significant main effect of Drug in 2-way ANOVA **e** possibly reflecting fewer high-bursting neurons **f-g**, post-hoc testing did not reveal significant changes in proportion of burst activity in either non-stressed or CMS-exposed rats treated repeatedly with quetiapine as compared to their respective control group treated repeatedly with vehicle

We next assessed whether repeated quetiapine administration differentially affected dopamine neuron activity in non-stressed and CMS-exposed rats. As with acute administration of quetiapine, we observed a significant interaction between CMS exposure and repeated treatment with quetiapine (*F*_(1,42)_ = 5.2, *p* = 0.028; Fig. 3, Table 2). However, in contrast to the effects of acute treatment, repeated quetiapine treatment did not alter dopamine neuron population activity in non-stressed rats at these doses (CON-VEH-Repeated 0.99 ± 0.06 CPT, *n* = 10; CON-QTP-Repeated 0.92 ± 0.10 CPT, *n* = 13; *t*_42_ = 0.53, *p* = 0.84; Table 2a, Fig. 3). Similarly, no effect was observed in average dopamine firing rate (CON-VEH-Repeated 2.9 ± 0.21 Hz, *n* = 78; CON-QTP-Repeated 3.4 ± 0.21 Hz, *n* = 92; 2-way ANOVA *p* > 0.05 for main effects of drug or interaction; Fig. 3b–d, Table 2). While a main effect of repeated quetiapine on proportion of burst activity was observed across all groups (F_(1,301)_ = 5.4; *p* = 0.020), a post-hoc test within non-stressed rats did not reveal a significant effect (CON-VEH-Repeated 31.6 ± 2.9%SIB, *n* = 78; CON-QTP-Repeated 23.8 ± 2.6%SIB, *n* = 92; *t*_(301)_ = 2.0; *p* = 0.084; Fig. 3e–g, Table 2).

**Table 2:** Summary of dopamine neuron data for repeated quetiapine treatment in Experiment 2

	CON-VEH-Repeated	CON-QTP-Repeated	CMS-VEH-Repeated	CMS-QTP-Repeated
**Cells Per Track**	**0.99** ± **0.060**	**0.92** ± **0.10**	**0.58** ± **0.070**	**0.93** ± **0.11**
2-Way interaction	**F**_**(1,42)**_ = **5.2;** ***p*** = **0.028** ^**a**^	**-**	-	**-**
Drug effect	F_(1,42)_ = 2.3; *p* = 0.13^a^	t_(42)_ = 0.53; *p* = 0.84^b^	-	**t**_**(42)**_ = **2.7;** ***p*** = **0.021** ^**b**^
Stress effect	**F**_**(1,42)**_ = **4.5;** ***p*** = **0.040** ^**a**^	-	**t**_**(42)**_ = **2.9;** ***p*** = **0.011** ^**c**^	t_(42)_ = 0.11; *p* = 0.99^c^
**Firing Rate (Hz)**	**2.9** ± **0.21**	**3.4** ± **0.21**	**3.4** ± **0.34**	**3.2** ± **0.21**
2-Way interaction	F_(1,42)_ = 1.5; *p* = 0.21^a^	-^d^	-^d^	**-** ^d^
Drug effect	F_(1,42)_ = 0.22; *p* = 0.64^a^	-^d^	-	-^d^
Stress effect	F_(1,42)_ = 0.23; *p* = 0.63^a^	-	-^d^	-^d^
**Bursting (%SIB)**	**31.6** ± **2.9**	**23.8** ± **2.6**	**28.9** ± **4.2**	**22.8** ± **2.4**
2-Way interaction	F_(1,301)_ = 0.077; *p* = 0.78^a^	-^d^	-^d^	-^d^
Drug effect	**F**_**(1,301)**_ = **5.4;** ***p*** = **0.020** ^**a**^	t_(301)_ = 2.0; *p* = 0.084^b^	-	t_(301)_ = 1.3; *p* = 0.33^b^
Stress effect	F_(1,301)_ = 0.36; *p* = 0.55^a^	-	-^d^	-^d^
**Group N (Rats)**	***N*** = **10 rats, 78 cells**	***N*** = **13 rats, 92 cells**	***N*** = **10 rats, 44 cells**	***N*** = **13 rats, 91 cells**

^a^Main effects among all groups

^b^Sidak’s post-hoc test for drug effect (within same stress category)

^c^Sidak’s post-hoc test for stress effect (within same drug group)

^d^Main effect not significant, post-hoc test not performed

%SIB, Percent of Spikes in Burst; *CMS* chronic mild stress, *CON* control, *QTP* quetiapine, *VEH* vehicle

Rats exposed to CMS that received 21+ days of vehicle injections showed reduced dopamine neuron population activity compared to non-stressed rats treated repeatedly with vehicle (CON-VEH-Repeated 0.99 ± 0.06 CPT, *n* = 10; CMS-VEH-Repeated 0.58 ± 0.07 CPT, *n* = 10; *t*_42_ = 2.9, *p* = 0.011; Fig. 3a, Table 2). Consistent with the acutely-treated vehicle groups, CMS-exposed rats treated repeatedly with vehicle were similar to non-stressed rats treated repeatedly with vehicle in average dopamine neuron firing rate (CON-VEH-Repeated 2.9 ± 0.21 Hz, *n* = 78; CMS-VEH-Repeated 3.4 ± 0.34 Hz, *n* = 44; 2-way ANOVA *p* > 0.05 for main effects of stress or interaction; Fig. 3b–d, Table 2) and proportion of burst activity (CON-VEH-Repeated 31.6 ± 2.9%SIB, *n* = 78; CMS-VEH-Repeated 28.9 ± 4.2 %SIB, *n* = 44; 2-way ANOVA *p* > 0.05 for main effects of stress or interaction; Fig. 3e–g, Table 2).

In rats exposed to CMS, repeated treatment with quetiapine increased dopamine neuron population activity compared to CMS-exposed rats that received repeated treatment with vehicle (CMS-VEH-Repeated 0.58 ± 0.07 CPT, *n* = 10; CMS-QTP-Repeated 0.93 ± 0.11 CPT, *n* = 13; *t*_42_ = 2.7, *p* = 0.021; Fig. 3a, Table 2). This increase reversed the CMS-induced attenuation of dopamine neuron population activity to levels identical to that of the non-stressed control rats. Compared to rats treated repeatedly with vehicle, the rats that received repeated treatment with quetiapine did not show significantly altered average dopamine neuron firing rate (CMS-VEH-Repeated 3.4 ± 0.34 Hz, *n* = 44; CMS-QTP-Repeated 3.2 ± 0.21 Hz, *n* = 91; 2-way ANOVA *p* > 0.05 for main effects of drug or interaction; Fig. 3b–d, Table 2) or proportion of burst activity (CMS-VEH-Repeated 28.9 ± 4.2%SIB, *n* = 44; CMS-QTP-repeated 22.8 ± 2.4 %SIB, *n* = 91; *t*_301_ = 1.3, *p* = 0.33; Fig. 3e–g, Table 2), despite a significant main effect of drug treatment across all groups for bursting (*F*_(1,301)_ = 5.4; *p* = 0.020). Histogram review suggests preserved firing rate distribution (Fig. 3c, d), and the possibility of fewer high-bursting neurons in both CMS and non-stressed rats following repeated quetiapine (Fig. 3f, g).

### Experiment 3: Repeated low-dose quetiapine does not induce depolarization block in normal rats

Repeated administration of antipsychotic drugs, including high antipsychotic doses of second generation agents such as quetiapine, have been shown to induce depolarization block following > 21 days of administration to normal rats^15^. However, the induction of depolarization block following lower antidepressant doses of quetiapine such as 10 mg/kg used at present has not been examined. Thus, for a portion of the non-stressed rats that received repeated treatment with quetiapine in Experiment 2, we tested for the presence of depolarization block at the conclusion of dopamine neuron recording. Following an initial recording of dopamine neurons from 6–9 tracks within the A10 region, a dose of apomorphine selective for pre-synaptic D2 receptors (40–80 µg/kg) was administered via the lateral tail vein (infusion verified by a decrease in firing rate of a single recorded dopamine neuron), and an additional 6–9 tracks of dopamine neuron recording was performed, allowing for pre- and post-apomorphine assessments of dopamine neuron population activity within the same animal. We observed no change in dopamine neuron population activity following apomorphine administration (Pre-APO 0.75 ± 0.11 CPT; Post-APO 0.79 ± 0.14 CPT; paired *t*_3_ = 1, *p* = 0.39), suggesting that depolarization block did not occur following repeated administration of 10 mg/kg quetiapine.

## Discussion

In this study, we found divergent effects of acute and repeated administration of quetiapine in non-stressed and CMS-exposed rats. While acute quetiapine increased dopamine population activity in non-stressed rats, it had no effect on CMS-exposed rats. Conversely, quetiapine administered repeatedly was uniquely capable of restoring normal levels of dopamine neuron population activity in CMS-exposed rats, but had no persisting effects on population activity in non-stressed rats. Furthermore, we show that the return to baseline levels of dopamine neuron population activity in non-stressed rats administered quetiapine for 21 days is unlikely to be due to the induction of depolarization block in VTA dopamine neurons.

Prior studies have administered quetiapine to CMS-exposed rats and observed behavioral effects consistent with an antidepressant action^17^. However, as far as we are aware, this is the first study to examine the electrophysiological impact of quetiapine on the dopamine system in rats subjected to an animal model of depression. Our results suggest that quetiapine has significantly different effects on the dopamine system in CMS-exposed rats as compared to its effects in non-stressed rats. These data add to existing literature on the dopamine system impact of acute and repeated quetiapine treatment in normal rats. These studies found that acute administration of quetiapine at doses of 10 mg/kg or greater will induce acute increases in population activity of the A10 (i.e., VTA) but not A9 (i.e., substantia nigra) dopamine neurons^15^. Moreover, the impact of repeated administration of quetiapine was examined at increasing dosages, and found to induce depolarization inactivation only when administered at doses of 40 mg/kg or greater^16^. This higher dose is more in line with the doses used clinically in the treatment of psychosis or acute mania (i.e., 600–800 mg), whereas the dose used in the treatment of depression (i.e., 300 mg) is more in line with the 10 mg/kg dose used in the present study, as measured by D2 receptor occupancy^18^.

Although behavioral confirmation of the anhedonic state would have been useful to corroborate the electrophysiological studies, no universally accepted behavioral assays are available. Thus, future studies should assess whether stress pre-exposure alters the impact of a one-time dose or repeated doses of quetiapine. Of note, prior studies have demonstrated the ability of quetiapine to prevent stress induced depressive behaviors^17^. However, whether quetiapine can rescue an existing depressive phenotype remains to be examined.

The mechanism through which quetiapine, like other D2RAs, increases dopamine neuron population activity is believed to occur via activation of striatal-midbrain feedback circuits, given that lesions of these feedback pathways block the antipsychotic drug-induced increase in dopamine population activity^25^. Similarly, the induction of depolarization block by high-dose antipsychotic drugs is also believed to occur via hyperactivation of these pathways, resulting in over-depolarization of the dopamine neuron membrane potential to the point of spike inactivation^24^. One plausible mechanism by which quetiapine or other D2RA medications could be effective as antidepressants is that, at the low doses used to treat depression, the drugs produce sufficient activation of these feedback pathways to restore dopamine population activity to normal but not sufficient to induce depolarization block. In contrast, the low level of striatal D2 receptor blockade produced by low doses of quetiapine is likely to be compensated homeostatically by increased D2 receptor number, increased dopamine synthesis, increased release, etc. As a result, the normalization of dopamine neuron population activity in the depressed state produced by quetiapine results in a greater restoration of phasic dopamine system responsivity to afferent drive^3^ compared to the low tonic level of D2 receptor blockade.

The principle brain region believed to impact VTA dopamine neuron population activity is the ventral pallidum (VP), as pre-infusion of the GABA-A receptor antagonist into the VP blocks the increase in dopamine population activity observed following acute administration of sertindole or haloperidol^26^. Thus, D2RAs are hypothesized to enhance accumbal inhibitory drive of the VP, releasing dopamine neurons from VP inhibition and subsequently increasing the number that are spontaneously active. We have shown previously that the VP is also a critical node in the pathway driving the CMS-induced hypodopaminergic state^12^. Therefore, these collective data lead to the intriguing possibility that convergent and counteracting effects on the VP is the mechanism of action of these D2RA medications’ antidepressant effects, with CMS increasing and quetiapine decreasing VP inhibitory tone over VTA dopamine neurons.

The approach of using subsets of D2RA medications for an antidepressant effect is a promising therapeutic mechanism. The present study focused only on quetiapine rather than other D2RAs such as haloperidol or clozapine because quetiapine has been shown to possess antidepressant effects clinically^27^. Moreover, the binding profile of quetiapine is much different than other D2RA antipsychotic drugs, even within the second generation (atypical) class^28^. Thus, quetiapine demonstrates a unique receptor occupancy curve wherein it binds fleetingly with high potency and rapid dissociation, rather than a prolonged occupancy seen with other compounds^29^. It is possible that these additional collective qualities may account for the unique ability of quetiapine to act as an antidepressant medication while haloperidol and other more traditional antipsychotic drugs are not effective as antidepressants. Of note, recent clinical data supports a dopamine enhancing mechanism for D2RA treatment in MDD^30^. It is worth mentioning, however, that quetiapine and its metabolites do have a number of effects on other receptors such as 5-HT1A, which likely also contribute to a major portion of its antidepressant actions^31^.

In conclusion, the present data support unique and divergent effects of acute and repeated administration of the D2RA quetiapine in non-stressed and chronically stress-exposed rodents. These data offer compelling evidence for further investigating the dopaminergic component of the therapeutic mechanism of select D2RA medications, and highlight the importance of conducting these studies using an animal model of depression in addition to normal rats.

## Electronic supplementary material

Figure S1

Table S1

Table S2
