# Supplementary material for: Divergent effects of acute and repeated quetiapine treatment on dopamine neuron activity in normal vs. chronic mild stress induced hypodopaminergic states
Source: Transl Psychiatry. 2017 Dec 11;7:1275. doi: 10.1038/s41398-017-0039-9 (PMC5802622; doi:10.1038/s41398-017-0039-9)
Supplement: Supplementary file 1 — Figure S1 [file 41398_2017_39_MOESM1_ESM.docx]

**Figure S1. Additional Dopamine Neuron Burst Properties in Rats Treated Acutely or Repeatedly with Quetiapine.** There was no significant effect of acute or repeated quetiapine treatment on A) Average duration of bursts, B) Average number of spikes per burst, C) Average firing rate within bursts, or D) Average Inter-Spike Interval (ISI) within bursts. E) Average Coefficient of Variation (CV) of the ISI within bursts was not significantly different for rats treated acutely with quetiapine. For rats treated repeatedly with quetiapine there was a significant interaction effect for CV of the ISI, however post-hoc analyses were not significant for any between group comparisons. See Tables S1 and S2 for results of all statistical analyses.
