# Supplementary material for: Divergent effects of acute and repeated quetiapine treatment on dopamine neuron activity in normal vs. chronic mild stress induced hypodopaminergic states
Source: Transl Psychiatry. 2017 Dec 11;7:1275. doi: 10.1038/s41398-017-0039-9 (PMC5802622; doi:10.1038/s41398-017-0039-9)
Supplement: Supplementary file 2 — Table S1 [file 41398_2017_39_MOESM2_ESM.docx]

**Table S1. Summary of Additional Dopamine Neuron Bursting Properties After Acute Quetiapine**

|  | **CON-VEH-Acute** | **CON-QTP-Acute** | **CMS-VEH-Acute** | **CMS-QTP-Acute** |
| --- | --- | --- | --- | --- |
| **Burst Duration (sec)** | **0.21 ± 0.05** | **0.13 ± 0.01** | **0.12 ± 0.02** | **0.11 ± 0.02** |
| 2-Way Interaction | F_(1,206)_ = 0.47; p = 0.49^a^ | -^d^ | -^d^ | -^d^ |
| Drug Effect | F_(1,206)_ = 1.1; p = 0.29^a^ | -^d^ | - | -^d^ |
| Stress Effect | F_(1,206)_ = 1.1; p = 0.30^a^ | - | -^d^ | -^d^ |
|  |  |  |  |  |
| **Spikes Per Burst** | **3.8 ± 0.63** | **3.0 ± 0.15** | **2.9 ± 0.21** | **2.7 ± 0.17** |
| 2-Way Interaction | F_(1,206)_ = 0.28; p = 0.60^a^ | -^d^ | -^d^ | -^d^ |
| Drug Effect | F_(1,206)_ = 0.98; p = 0.33^a^ | -^d^ | - | -^d^ |
| Stress Effect | F_(1,206)_ = 1.2; p = 0.27^a^ | - | -^d^ | -^d^ |
|  |  |  |  |  |
| **Burst ISI (sec)** | **0.069 ± 0.0020** | **0.062 ± 0.0017** | **0.066 ± 0.0030** | **0.065 ± 0.0033** |
| 2-Way Interaction | F_(1,206)_ = 0.91 ; p = 0.34^a^ | -^d^ | -^d^ | -^d^ |
| Drug Effect | F_(1,206)_ = 0.95; p = 0.33^a^ | -^d^ | - | -^d^ |
| Stress Effect | F_(1,206)_ = 0.029; p = 0.86^a^ | - | -^d^ | -^d^ |
|  |  |  |  |  |
| **Burst CV** | **0.42 ± 0.024** | **0.41 ± 0.020** | **0.38 ± 0.032** | **0.41 ± 0.030** |
| 2-Way Interaction | F_(1,206)_ = 0.28 ; p = 0.59^a^ | -^d^ | -^d^ | -^d^ |
| Drug Effect | F_(1,206)_ = 0.041; p = 0.84^a^ | -^d^ | - | -^d^ |
| Stress Effect | F_(1,206)_ = 0.44; p = 0.51^a^ | - | -^d^ | -^d^ |
|  |  |  |  |  |
| **Burst Firing Rate (Hz)** | **17.8 ± 0.78** | **20.7 ± 1.0** | **17.6 ± 0.94** | **18.6 ± 0.99** |
| 2-Way Interaction | F_(1,206)_ = 0.42 ; p = 0.52^a^ | -^d^ | -^d^ | -^d^ |
| Drug Effect | F_(1,206)_ = 1.6; p = 0.21^a^ | -^d^ | - | -^d^ |
| Stress Effect | F_(1,206)_ = 0.61; p = 0.44^a^ | - | -^d^ | -^d^ |
|  |  |  |  |  |
| **Group N (Rats)** | **N = 8 rats, 64 cells** | **N = 11 rats, 108 cells** | **N = 4 rats, 18 cells** | **N = 6 rats, 20 cells** |

^a^Main effects among all groups;

^b^Sidak’s post-hoc test for drug effect (within same stress category);

^c^Sidak’s post-hoc test for stress effect (within same drug group);

^d^Main effect not significant, post-hoc test not performed

CMS, Chronic Mild Stress; CON, Control; CV, Coefficient of Variation; ISI, Inter-Spike Interval; QTP, Quetiapine; VEH, Vehicle
