# Supplementary material for: Divergent effects of acute and repeated quetiapine treatment on dopamine neuron activity in normal vs. chronic mild stress induced hypodopaminergic states
Source: Transl Psychiatry. 2017 Dec 11;7:1275. doi: 10.1038/s41398-017-0039-9 (PMC5802622; doi:10.1038/s41398-017-0039-9)
Supplement: Supplementary file 3 — Table S2 [file 41398_2017_39_MOESM3_ESM.docx]

**Table S2. Summary of Additional Dopamine Neuron Bursting Properties After Repeated Quetiapine**

|  | **CON-VEH-Acute** | **CON-QTP-Acute** | **CMS-VEH-Acute** | **CMS-QTP-Acute** |
| --- | --- | --- | --- | --- |
| **Burst Duration (sec)** | **0.12 ± 0.02** | **0.13 ± 0.01** | **0.12 ± 0.01** | **0.12 ± 0.01** |
| Interaction | F_(1,276)_ = 0.00035; p = 0.98^a^ | -^d^ | -^d^ | -^d^ |
| Drug | F_(1,276)_ = 0.013; p = 0.91^a^ | -^d^ | - | -^d^ |
| Stress | F_(1,276)_ = 0.56; p = 0.45^a^ | - | -^d^ | -^d^ |
|  |  |  |  |  |
| **Spikes Per Burst** | **3.1 ± 0.22** | **3.1 ± 0.18** | **2.9 ± 0.19** | **3.0 ± 0.09** |
| Interaction | F_(1,276)_ = 0.027; p = 0.87^a^ | -^d^ | -^d^ | -^d^ |
| Drug | F_(1,276)_ = 0.0064; p = 0.94^a^ | -^d^ | - | -^d^ |
| Stress | F_(1,276)_ = 0.40; p = 0.53^a^ | - | -^d^ | -^d^ |
|  |  |  |  |  |
| **Burst ISI (sec)** | **0.058 ± 0.0022** | **0.062 ± 0.0022** | **0.059 ± 0.0033** | **0.056 ± 0.0025** |
| Interaction | F_(1,276)_ = 1.7 ; p = 0.19^a^ | -^d^ | -^d^ | -^d^ |
| Drug | F_(1,276)_ = 0.088; p = 0.77^a^ | -^d^ | - | -^d^ |
| Stress | F_(1,276)_ = 0.90; p = 0.34^a^ | - | -^d^ | -^d^ |
|  |  |  |  |  |
| **Burst CV** | **0.51 ± 0.022** | **0.44 ± 0.024** | **0.43 ± 0.040** | **0.49 ± 0.032** |
| Interaction | **F_(1,276)_ = 4.5 ; p = 0.035^a^** | t_(276)_ = 1.6; p = 0.20^b^ | t_(276)_ = 1.7; p = 0.16^c^ | t_(276)_ = 1.4; p = 0.30^b^ |
| Drug | F_(1,276)_ = 0.00032; p = 0.99^a^ | -^d^ | - | -^d^ |
| Stress | F_(1,276)_ = 0.37; p = 0.55^a^ | - | -^d^ | -^d^ |
|  |  |  |  |  |
| **Burst Firing Rate (Hz)** | **23.2 ± 1.3** | **22.1 ± 1.4** | **24.3 ± 2.4** | **26.2 ± 1.7** |
| Interaction | F_(1,276)_ = 0.80 ; p = 0.37^a^ | -^d^ | -^d^ | -^d^ |
| Drug | F_(1,276)_ = 0.045; p = 0.83^a^ | -^d^ | - | -^d^ |
| Stress | F_(1,276)_ = 2.3; p = 0.13^a^ | - | -^d^ | -^d^ |
|  |  |  |  |  |
| **Group N (Rats)** | **N = 10 rats, 73 cells** | **N = 13 rats, 81 cells** | **N = 10 rats, 42 cells** | **N = 13 rats, 84 cells** |

^a^Main effects among all groups;

^b^Sidak’s post-hoc test for drug effect (within same stress category);

^c^Sidak’s post-hoc test for stress effect (within same drug group);

^d^Main effect not significant, post-hoc test not performed

CMS, Chronic Mild Stress; CON, Control; CV, Coefficient of Variation; ISI, Inter-Spike Interval; QTP, Quetiapine; VEH, Vehicle
